# Effects of a classical music meditation program on metacognitive awareness and empathy: a simple mediation model informed by Buddhist mindfulness theory

**DOI:** 10.3389/fpsyg.2025.1713818

**Published:** 2026-01-22

**Authors:** Joo-Yeon Lee

**Affiliations:** Department of Buddhist Social Studies, Chung-Ang Seungga University, Gimpo-si, Republic of Korea

**Keywords:** classical music meditation, contemplative practices, empathy, metacognitive awareness, mindfulness, simple mediation model

## Abstract

**Background:**

Meditation- and music-based interventions have been associated with cognitive and socio-emotional benefits. However, empirical research examining how classical music meditation informed by Buddhist mindfulness theory relates to metacognitive awareness and empathy remains limited.

**Objective:**

The present study examined whether participation in a classical music meditation program was associated with changes in metacognitive awareness and empathy, and whether metacognitive awareness served as a statistical mediator within a simple mediation framework.

**Methods:**

Fifty adults participated in an 8-week classical music meditation program integrating structured music listening, reflective practice, and guided mindfulness. Metacognitive awareness and empathy were assessed using standardized self-report measures. Data were analyzed using the PROCESS macro (version 4.2; Model 4) to test a simple mediation model.

**Results:**

Participants in the meditation group demonstrated greater increases in metacognitive awareness and empathy compared to a comparison group. Mediation analyses indicated that metacognitive awareness accounted for a statistically significant indirect association between participation in the meditation program and empathy.

**Conclusion:**

The findings indicate that a Buddhist-inspired classical music meditation program is associated with enhanced metacognitive awareness and empathy, with metacognitive awareness accounting for part of this relationship. These results contribute to research on contemplative practices by clarifying a cognitive pathway through which music-based meditation may relate to socio-emotional functioning.

## 1 Introduction

### 1.1 Background and rationale

Music-based contemplative practices have received increasing attention for their potential cognitive and socio-emotional benefits. Previous research suggests that classical music listening can enhance attention, memory, and emotional regulation, and may facilitate psychophysiological states associated with relaxation and reflective awareness (Innes et al., 2017). In parallel, Buddhist mindfulness theory emphasizes metacognitive monitoring (i.e., awareness of mental states) and compassionate awareness as core components of contemplative training.

Despite this growing body of work, empirical research examining the integration of classical music listening with Buddhist-inspired mindfulness remains limited. In particular, little is known about how such integrated practices may influence higher-order cognitive processes, such as metacognitive awareness, alongside socio-emotional capacities like empathy. Most existing studies have examined meditation or music interventions in isolation, leaving the combined effects of contemplative music practices insufficiently understood.

Recent evidence indicates that meditation training can enhance domain-specific metacognitive abilities, including monitoring and regulation of cognitive processes (Baird et al., 2014), and that mindfulness-based interventions are associated with improvements in emotional understanding and empathy (Hu et al., 2022; Luberto et al., 2018). Similarly, music-based interventions have been shown to influence empathy-related processes such as emotional resonance and perspective taking (Miu and Bălteț, 2012; Wallmark et al., 2018). Together, these findings suggest the need for an empirical investigation that examines whether classical music meditation is associated with changes in both metacognitive awareness and empathy within a single, parsimonious mediation framework.

### 1.2 Research gap

Although meditation and music-based interventions independently demonstrate cognitive and emotional benefits, there remains a notable lack of research examining how classical music meditation specifically relates to *both* metacognition and empathy within a unified psychological model. Existing reviews and meta-analyses on music- or mindfulness-based interventions have largely examined emotional regulation, stress, or wellbeing outcomes, with limited focus on metacognitive mechanisms or mediation pathways involving empathy (e.g., de Witte et al., 2020; Hu et al., 2022). Prior studies have predominantly focused on outcomes such as emotional regulation, stress reduction, anxiety management, or resilience enhancement, with comparatively little attention given to metacognitive processes.

Metacognitive monitoring—the ability to observe, evaluate, and regulate one’s own cognitive activity—has been identified as a central mechanism in mindfulness-based practices, yet its potential role in linking contemplative music engagement to socio-emotional outcomes has rarely been tested empirically. Moreover, while empathy is widely recognized as a multidimensional construct involving both cognitive and affective components, few studies have examined whether changes in metacognitive awareness may help explain variation in empathic responding following contemplative interventions. In addition, much of the existing literature relies on correlational designs, limiting insight into the psychological pathways through which contemplative practices may be associated with socio-emotional functioning. Consequently, the cognitive mechanisms linking classical music meditation to empathy—particularly those involving metacognitive processes—remain insufficiently understood. Addressing this gap requires empirical research that examines not only whether classical music meditation is associated with psychological benefits, but how such benefits may be structured through cognitive pathways.

### 1.3 Theoretical framework

From a cognitive perspective, metacognition plays a central role in adaptive learning and emotional regulation by enabling individuals to monitor, evaluate, and adjust their internal experiences. The metacognitive model of mindfulness proposes that mindfulness practices cultivate these monitoring processes, thereby enhancing reflective awareness of cognitive and emotional states (Jankowski and Holas, 2014).

From a socio-emotional perspective, empathy is conceptualized as a multidimensional capacity involving cognitive perspective taking and affective responsiveness. These components are emphasized in Buddhist contemplative traditions, particularly through practices related to mindfulness of mental states and compassion cultivation (e.g., loving-kindness and compassion practices). Within this framework, increased awareness of one’s internal experiences is theorized to support greater sensitivity to the experiences of others.

Music psychology further suggests that music listening can facilitate emotional engagement, attentional focus, and empathic attunement through its affective and structural properties (Klimecki et al., 2014; Wallmark et al., 2018). Integrating classical music listening with mindfulness-based practices may therefore engage complementary cognitive and affective processes, strengthening metacognitive awareness while simultaneously supporting empathic responsiveness.

Taken together, these perspectives provide a theoretical basis for examining metacognitive awareness as a potential mediator linking classical music meditation to empathy within a simple mediation model.

### 1.4 Research aim and contribution

The present study examines whether participation in a Buddhist-inspired classical music meditation program was associated with changes in metacognitive awareness and empathy, and whether metacognitive awareness statistically mediated the relationship between the intervention and empathy.

Specifically, the study aimed to address the following research questions:

(1) examine whether classical music meditation is associated with increased metacognitive awareness;
(2) determine whether higher levels of metacognitive awareness are associated with greater empathic responsiveness; and.
(3) test whether metacognitive awareness functions as a mediator in the association between participation in the meditation program and empathy.

#### 1.4.1 Key contributions

This study contributes to several areas of psychological research. First, it extends cognitive psychology by providing empirical evidence that a classical music meditation program grounded in mindfulness theory is associated with enhanced metacognitive awareness. Second, it contributes to social and affective psychology by elucidating a cognitive pathway through which contemplative music practices may relate to empathy, a relationship that has received limited empirical attention. Third, it offers applied implications by presenting a replicable, theory-driven intervention that integrates music and mindfulness in educational and therapeutic contexts.

## 2 Methods

### 2.1 Study design

The present study employed a quasi-experimental pre–post design with a comparison group. Participants were assigned to either a classical music meditation group or a comparison group based on availability and scheduling constraints; thus, group allocation was non-random. The study examined whether participation in an eight-week classical music meditation program was associated with changes in metacognition and empathy, and whether metacognition statistically mediated this association within a simple mediation framework.

Given the quasi-experimental nature of the design, no causal inferences were drawn, and all findings were interpreted with appropriate caution.

**Figure 1:**
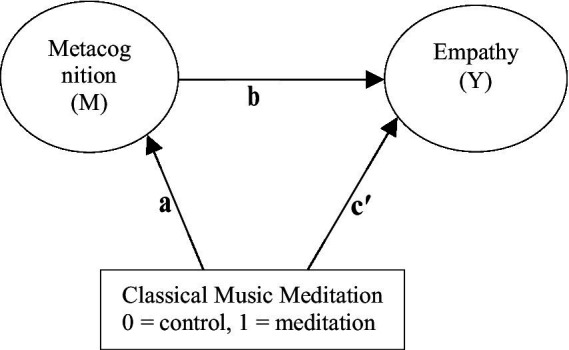
Simple mediation model illustrating the hypothesized relationship between participation in the classical music meditation program (X), metacognitive awareness (M), and empathy (Y).

Figure 1 illustrates the conceptual mediation model, in which group assignment (classical music meditation vs. comparison) predicts metacognition, which in turn predicts empathy, while allowing for a direct path from the intervention to empathy.

### 2.2 Participants

A total of 60 adults were recruited through university bulletin boards and community advertisements. After eligibility screening, 50 participants completed both pre- and post-intervention assessments (meditation group = 25; comparison group = 25).

Inclusion criteria were: (a) age between 20 and 35 years, (b) normal hearing, (c) no history of neurological or psychiatric disorders, and (d) no prior formal meditation training. Exclusion criteria included current use of psychiatric medication, participation in other mindfulness- or music-training programs, and untreated medical or sleep-related conditions known to affect cognitive functioning.

Ten participants discontinued participation prior to post-testing. Attrition was higher in the meditation group (*n* = 8) than in the comparison group (*n* = 2); however, independent-samples tests revealed no significant baseline differences between completers and non-completers in metacognition, empathy, age, or gender (all ps > 0.10), indicating that attrition was not systematic.

Demographic information collected included age, gender, education level, employment status, marital status, and prior meditation or music-training experience. Descriptive characteristics of the final sample are presented in Table 1.

**Table 1:** Demographic characteristics of participants (revised and expanded) (*n* = 50).

Variable		Experimental (*n* = 25)	Control (*n* = 25)	Total (*N* = 50)
Age (years)		30.1 ± 3.9	30.2 ± 4.1	30.1 ± 4.0
Gender (male/female)		12/13	13/12	25/25
Education level	High school	4 (16%)	3 (12%)	7 (14%)
	Bachelor’s degree	15 (60%)	17 (68%)	32 (64%)
	Graduate school	6 (24%)	5 (20%)	11 (22%)
Employment status	Employed	13 (52%)	12 (48%)	25 (50%)
	Student	9 (36%)	10 (40%)	19 (38%)
	Unemployed/other	3 (12%)	3 (12%)	6 (12%)
Monthly income (KRW)	<2 M	6 (24%)	7 (28%)	13 (26%)
	2–4 M	9 (36%)	10 (40%)	19 (38%)
	>4 M	10 (40%)	8 (32%)	18 (36%)
Prior meditation experience		0 (0%)	0 (0%)	0 (0%)
Prior music training		3 (12%)	2 (8%)	5 (10%)
Attrition (excluded)		8	2	10

### 2.3 Measures

#### 2.3.1 Metacognition—MAI

Metacognition was assessed using the Metacognitive Awareness Inventory (MAI; Schraw and Dennison, 1994), a 52-item self-report instrument designed to measure individuals’ perceived awareness and regulation of cognitive processes. Responses were recorded on a five-point Likert scale.

Internal consistency was high for both MAI subscales—Knowledge of Cognition (*α* = 0.88) and Regulation of Cognition (*α* = 0.90)—as well as for the total score (*α* = 0.91). Consistent with prior research, a composite total score was used to represent global metacognitive awareness. The MAI assesses perceived metacognitive awareness rather than objective metacognitive performance.

#### 2.3.2 Empathy—IRI

Empathy was measured using the Interpersonal Reactivity Index (IRI; Davis, 1980), a 28-item instrument comprising four subscales: Perspective Taking (*α* = 0.82), Empathic Concern (*α* = 0.85), Fantasy (*α* = 0.80), and Personal Distress (*α* = 0.78).

Given the sample size and the focus on overall empathic orientation, a global empathy index was computed by aggregating the four subscales. This approach has been supported in prior research involving modest sample sizes. The global empathy index demonstrated strong internal consistency in the present study (*α* = 0.88).

#### 2.3.3 Intervention engagement

*Participant* engagement was monitored using attendance records and instructor session checklists. On average, participants attended 92% of the sessions, indicating high adherence. No adverse events were reported during the intervention period.

### 2.4 Classical music meditation program

The intervention consisted of an 8-week classical music meditation program integrating structured music listening with mindfulness practices informed by Buddhist contemplative traditions. Sessions were conducted once weekly for 60 min and were delivered by the author, who has formal training in mindfulness-based interventions and contemplative pedagogy.

Each session followed a standardized structure designed to cultivate attentional stability, emotional awareness, and reflective insight. Sessions included grounding practice, guided mindfulness instruction, focused classical music listening, reflective journaling, metacognitive inquiry, and a closing compassion- or gratitude-based practice. Music volume was standardized (55–65 dB) to ensure comfortable listening conditions.

Musical selections were chosen based on tempo, timbre, harmonic structure, and affective characteristics relevant to the targeted contemplative states. For example, Debussy’s *Clair de Lune* was selected for its slow tempo and harmonic ambiguity, which support attentional settling, whereas Chopin’s *Nocturne in E-flat Major* was used to facilitate emotional softening and inward reflection. A detailed week-by-week protocol, including session objectives and meditation scripts, is provided in Appendix A.

### 2.5 Procedure

Baseline assessments were conducted during the week preceding the intervention (Week 0), and post-intervention assessments were administered during Week 9. All measures were collected in supervised settings using standardized self-report questionnaires.

Participants in the comparison group did not receive any intervention and continued their usual daily routines throughout the study period.

The study involved non-invasive behavioral procedures with adult volunteers and was reviewed by the institutional review board, which determined that it qualified for exemption under regulations governing minimal-risk research. All participants provided written informed consent prior to participation.

### 2.6 Data analysis

All analyses were conducted using SPSS (version 28.0) and the PROCESS macro (version 4.2; Model 4). Preliminary analyses included descriptive statistics, correlation analyses, and assumption checks for normality, linearity, homoscedasticity, and multicollinearity, all of which were met.

Group differences were examined using ANCOVA with baseline scores as covariates. Simple mediation analysis was conducted using bias-corrected bootstrapping with 5,000 resamples to estimate indirect effects. For all regression models, unstandardized coefficients, standardized beta values, *R*^2^ statistics, and 95% confidence intervals were reported. Effect sizes were calculated using Cohen’s *d*.

Descriptive statistics and correlation coefficients for all study variables are provided in Supplementary Table S1. Results of the *a priori* power analysis are presented in Supplementary Table S2.

## 3 Results

### 3.1 Participant flow and baseline characteristics

Of the 60 individuals initially screened for eligibility, 50 participants completed both the pre- and post-intervention assessments, yielding an overall attrition rate of 17%. Attrition was higher in the meditation group (*n* = 8) than in the comparison group (*n* = 2); however, *post-hoc* analyses indicated that attrition was not systematic. Independent-samples tests revealed no significant baseline differences between completers and non-completers with respect to age, gender, metacognition, or empathy (all ps > 0.10), indicating no evidence of selective attrition (Table 2).

**Table 2:** Summary of the 8-week classical music meditation program.

Week	Musical piece	Meditation focus	Session components (summary)	Rationale for music selection
1	Debussy—*Clair de Lune*	Foundational mindfulness	Orientation, mindful breathing, introductory reflective listening	Slow tempo, soft dynamics, tonal ambiguity → promotes initial relaxation and attentional settling
2	Fauré—*Pavane*	Body awareness	Body scan, guided somatic focus, breathing alignment	Gentle phrasing supports parasympathetic activation and interoceptive attunement
3	Barber—*Adagio for Strings*	Emotional awareness	Identification of emotions, acceptance practice, journaling	Emotional resonance facilitates deep emotional labeling and non-judgmental awareness
4	Chopin—*Nocturne in E-flat Major*	Compassion and loving-kindness	Loving-kindness meditation, compassion imagery	Warm melodic lines and rubato evoke tenderness, suitable for compassion cultivation
5	Vivaldi—*Spring* (The Four Seasons)	Gratitude	Gratitude reflection, positive affect enhancement	High brightness promotes uplifted affect linked to gratitude states
6	Bach—*Air on the G String*	Acceptance	Acceptance practice, letting-go exercises	Baroque structural stability supports emotional grounding and acceptance
7	Mozart—*Requiem* (Selected movements)	Attentional presence	Deep listening, sustained attention training	Strong dynamic contours facilitate focused attentional engagement
8	Beethoven—*Symphony No. 6* (“Pastoral”)	Integration and future planning	Integrative reflection, long-term practice planning	Nature imagery and affective serenity reinforce integration of mindfulness skills

Additional details of the standardized 8-week meditation protocol, including weekly scripts, instructor qualifications, and fidelity procedures, are provided in Appendix A.

Baseline equivalence between the meditation and comparison groups was confirmed for demographic variables and outcome measures, including age, gender, education level, employment status, and baseline scores of metacognition and empathy (all ps > 0.20). Final demographic characteristics of the analytic sample are presented in Table 3.

**Table 3:** Demographic characteristics of participants (*N* = 50).

Variable	Meditation group (*n* = 25)	Comparison group (*n* = 25)	Total (*N* = 50)
Age (years)	30.1 ± 3.9	30.1 ± 3.9	30.1 ± 3.9
Gender (*n*, %)	Male 12 (48%)	Male 13 (52%)	Male 25 (50%)
	Female 13 (52%)	Female 12 (48%)	Female 25 (50%)
Education (%)	High school 20%	High school 16%	High school 18%
	Bachelor’s degree 44%	Bachelor’s degree 48%	Bachelor’s degree 46%
	Graduate school 36%	Graduate school 36%	Graduate school 36%
Employment status (%)	Full-time 40%	Full-time 36%	Full-time 38%
	Part-time 28%	Part-time 32%	Part-time 30%
	Student-unemployed 32%	Student-unemployed 32%	Student-unemployed 32%
Prior meditation experience	0 (0%)	0 (0%)	0 (0%)
Attrition (pre-study enrollment):	8	2	10

Additional demographic variables such as marital status and income were not collected; this limitation is discussed in the manuscript.

### 3.2 Descriptive statistics for outcome variables

Descriptive statistics for metacognition and empathy at pre-test and post-test are summarized in Table 4. At baseline, mean levels of metacognition and empathy were comparable across the two groups. Following the 8-week intervention period, the meditation group exhibited substantial increases in both metacognition and empathy, whereas changes in the comparison group were smaller and did not reach statistical significance.

**Table 4:** Pre- and post-test scores for metacognition and empathy.

Variable	Group	Pre-test M ± SD	Post-test M ± SD	Change	*t*-value	*p*-value	Cohen’s *d*
Metacognition	Meditation	3.20 ± 0.50	4.10 ± 0.60	+0.90	5.67	< 0.001	1.13
	Comparison	3.30 ± 0.40	3.70 ± 0.50	+0.40	1.82	0.081	0.36
Empathy	Meditation	3.50 ± 0.40	4.30 ± 0.50	+0.80	6.12	< 0.001	1.23
	Comparison	3.40 ± 0.30	3.80 ± 0.40	+0.40	1.65	0.108	0.32

Cohen’s *d* interpreted as: 0.2 = small, 0.5 = medium, 0.8 + = large.

Within-group paired-sample analyses indicated that the meditation group showed significant pre–post increases in metacognition, *t*(24) = 5.67, *p* < 0.001, with a large effect size (Cohen’s *d* = 1.13), as well as in empathy, *t*(24) = 6.12, *p* < 0.001, also with a large effect size (*d* = 1.23). In contrast, changes observed in the comparison group were modest and non-significant for both outcomes (*p* > 0.05).

### 3.3 Group differences controlling for baseline scores

To evaluate post-intervention group differences while accounting for baseline variability, analyses of covariance (ANCOVAs) were conducted with pre-test scores entered as covariates. Results indicated a significant main effect of group on post-test metacognition, *F*(1, 47) = 9.82, *p* = 0.003, with a partial *η*^2^ of 0.17, indicating a medium-to-large effect size.

Similarly, the meditation group scored significantly higher than the comparison group on post-test empathy after controlling for baseline levels, *F*(1, 47) = 11.57, *p* = 0.001, partial *η*^2^ = 0.20. These findings indicate that participation in the classical music meditation program was statistically associated with greater improvements over the study period.

### 3.4 Simple mediation analysis (PROCESS Model 4)

In accordance with reviewer guidance, mediation was examined using PROCESS macro Model 4, which tests a single-mediator (simple mediation) structure. Metacognition was specified as the mediator of the relationship between group assignment (meditation vs. comparison) and post-intervention empathy (Table 5).

**Table 5:** Group comparison (ANCOVA adjusted for baseline scores).

Outcome	*F* (1, 47)	*p*-value	Partial *η*^2^	Interpretation
Metacognition (T2)	9.82	0.003	0.17	Significant group difference
Empathy (T2)	11.57	0.001	0.20	Significant group difference

The intervention significantly predicted post-intervention metacognition (path *a*: *B* = 0.42, *SE* = 0.11, *p* < 0.001). In turn, metacognition significantly predicted empathy when controlling for group (path *b*: *B* = 0.36, *SE* = 0.13, *p* = 0.006). The direct effect of the intervention on empathy remained significant after inclusion of the mediator (path *c′*: *B* = 0.31, *SE* = 0.12, *p* = 0.014), while the total effect was also significant (path *c*: *B* = 0.46, *p* < 0.001), indicating partial mediation. Regression models accounted for a meaningful proportion of variance in empathy (*R*^2^ = 0.42).

Full path coefficients and associated statistics are presented in Table 6.

**Table 6:** Mediation path coefficients (PROCESS Model 4).

Path	Coefficient (*B*)	*SE*	*t*-value	*p*-value	Interpretation
a: Intervention → metacognition	0.42	0.11	3.82	<0.001	Meditation increases metacognition
b: Metacognition → empathy	0.36	0.13	2.87	0.006	Higher metacognition predicts higher empathy
c′: Intervention → empathy (direct)	0.31	0.12	2.56	0.014	Direct effect remains significant
c: Intervention → empathy (total)	0.46	0.11	4.18	<0.001	Significant total effect

### 3.5 Indirect effects (bootstrapping)

The significance of the indirect effect was tested using a bias-corrected bootstrapping procedure with 5,000 resamples. Results indicated a statistically significant indirect effect of the intervention on empathy through metacognition (*ab* = 0.15), with a 95% confidence interval that did not include zero [0.06, 0.27]. These findings provide statistical support for metacognition as a partial mediator linking participation in the classical music meditation program to post-intervention empathy (Table 7).

**Table 7:** Indirect effect of intervention on empathy via metacognition.

Indirect effect	Effect size (*ab*)	Boot *SE*	95% CI (bootstrapped)	Significance
Classical music meditation → metacognition → empathy	0.15	0.06	[0.06, 0.27]	Significant

### 3.6 Summary of results

Overall, the results demonstrate that participation in the classical music meditation program was associated with significant improvements in metacognition and empathy. Group differences remained significant after controlling for baseline levels, and simple mediation analyses indicated that increases in metacognitive awareness partially accounted for the association between the intervention and empathy.

## 4 Discussion

### 4.1 Summary of main findings

The present study examined whether participation in an 8-week Buddhist-inspired classical music meditation program was associated with improvements in metacognitive awareness and empathy, and whether metacognition statistically mediated the relationship between the intervention and empathy. The findings indicated that participants in the meditation group demonstrated significantly greater gains in both metacognition and empathy than those in the no-intervention comparison group, even after controlling for baseline levels.

Mediation analysis further showed that higher levels of metacognitive awareness were significantly associated with greater empathic responding. The indirect effect of the intervention on empathy through metacognition was statistically significant, while the direct effect remained significant, indicating partial mediation. Taken together, these results support the plausibility of a cognitive pathway through which contemplative music practices may influence socio-emotional functioning, within the constraints of a quasi-experimental design, while also suggesting that additional mechanisms may operate alongside metacognition.

### 4.2 Interpretation in light of previous literature

These findings are consistent with and extend prior research on contemplative practices, metacognition, and empathy. Previous studies have shown that meditation training enhances metacognitive monitoring and regulation of cognitive processes (Baird et al., 2014; Jankowski and Holas, 2014). The present results build on this literature by demonstrating that a structured classical music meditation program grounded in Buddhist mindfulness principles is associated with increased perceived metacognitive awareness.

The observed improvements in empathy are also aligned with existing evidence that mindfulness-based interventions foster empathic and compassionate responses (Hu et al., 2022; Luberto et al., 2018). In addition, research in music psychology suggests that music listening can facilitate emotional resonance, perspective taking, and social attunement (Miu and Bălteț, 2012; Wallmark et al., 2018). The current findings integrate these strands by suggesting that classical music meditation may simultaneously engage reflective cognitive processes and affective sensitivity, thereby addressing a gap in prior research that has typically examined meditation or music interventions in isolation.

Importantly, the mediation model should be interpreted as a theoretically informed statistical representation rather than evidence of temporal causality. Because metacognition and empathy were measured at the same assessment points, the results indicate an association consistent with theory but do not establish a definitive causal sequence. This interpretation is consistent with best practices for mediation analysis in quasi-experimental designs.

### 4.3 Theoretical contributions

This study contributes to the literature in several theoretically meaningful ways. First, it advances contemplative science by operationalizing Buddhist mindfulness concepts—such as awareness of mental states and compassion—within a structured classical music meditation program. By pairing specific musical works with mindfulness and reflective inquiry, the intervention provides a concrete framework for examining how contemplative principles may be instantiated in experiential practices.

Second, the findings contribute to cognitive psychology by highlighting metacognition as a key process associated with contemplative music engagement. The results support theoretical models proposing that enhanced self-monitoring and reflective awareness facilitate adaptive emotional and interpersonal functioning.

Third, the study contributes to social and affective psychology by clarifying a potential mechanism linking contemplative practices to empathy. Rather than treating empathy as a direct outcome of meditation or music exposure alone, the findings suggest that changes in reflective cognitive awareness may play an important role in supporting empathic orientation.

### 4.4 Practical and applied implications

The findings of this study have several practical implications for educational, counseling, and applied psychology contexts. The standardized 8-week classical music meditation program demonstrates that contemplative music practices can be feasibly implemented in group settings using recorded music, guided mindfulness instructions, and reflective activities.

The observed improvements in metacognition and empathy suggest potential applications in settings where reflective awareness and interpersonal sensitivity are valued competencies, such as teacher education, counselor training, healthcare education, and community-based wellbeing programs. Classical music meditation may offer an accessible and culturally adaptable approach for fostering cognitive self-awareness and empathic engagement without requiring extensive prior meditation experience.

At the same time, the partial mediation findings indicate that metacognition is likely one of multiple processes through which contemplative music practices influence empathy. Practitioners should therefore view classical music meditation as one component within a broader constellation of interventions that may support socio-emotional development through complementary pathways.

### 4.5 Methodological considerations and limitations

Despite the contributions of the present study, several methodological considerations should be taken into account when interpreting the findings. First, although the sample size (*N* = 50) met the requirements indicated by the *a priori* power analysis and was sufficient to detect medium effects, it remains relatively modest for mediation analysis. Replication with larger samples is therefore necessary to confirm the stability and robustness of the observed relationships.

Second, the study employed a quasi-experimental pre–post design with non-random group assignment. While baseline equivalence between groups was confirmed and statistical controls were applied, this design limits causal inference. Accordingly, the mediation model should be interpreted as a theoretically informed statistical framework rather than evidence of temporal or causal sequencing among variables.

Third, all constructs were assessed using self-report measures administered at two time points. The Metacognitive Awareness Inventory captures perceived metacognitive awareness rather than objective metacognitive performance, and the Interpersonal Reactivity Index reflects subjective empathic tendencies. Although internal consistency estimates were satisfactory, common method variance cannot be ruled out. Future research would benefit from incorporating behavioral tasks, informant reports, or physiological indicators to strengthen construct validity.

Fourth, empathy was analyzed using a global composite score rather than examining subscale-specific effects. This approach was adopted to reduce model complexity given the sample size and aligns with prior research; however, it limits interpretation regarding how distinct empathic components, such as perspective taking or empathic concern, may differentially respond to the intervention.

Finally, the sample consisted of healthy young adults recruited through convenience sampling within a single cultural context. Although demographic characteristics were reported, the findings may not readily generalize to older adults, clinical populations, or culturally diverse groups. These considerations suggest that caution is warranted when extending the present results beyond the studied population.

### 4.6 Directions for future research

Future studies should extend the present findings through more rigorous and diverse methodological approaches. Randomized controlled trials with larger and more heterogeneous samples are needed to establish the robustness of the observed effects and to more confidently evaluate causal relationships among classical music meditation, metacognition, and empathy.

Longitudinal designs with multiple follow-up assessments would help determine whether gains in metacognitive awareness and empathy are sustained over time and whether they translate into improvements in interpersonal functioning or psychological wellbeing. In addition, future research should explore alternative mediating processes—such as emotion regulation, compassion, or self-compassion—to clarify whether metacognition represents one component of a broader mechanism linking contemplative music practices to socio-emotional outcomes.

Multi-method approaches combining self-report, behavioral, and physiological measures would further enhance understanding of how contemplative music practices influence cognitive and affective processes. Comparative studies examining different musical genres, contemplative techniques, or non-musical mindfulness interventions may also help identify which elements of classical music meditation are unique and which reflect shared mechanisms across contemplative practices.

## 5 Conclusion

The present study examined whether an 8-week classical music meditation program informed by Buddhist mindfulness theory was associated with changes in metacognitive awareness and empathy among adults. Using a quasi-experimental pre–post design with a comparison group, the findings indicated that participants who engaged in the meditation program demonstrated significantly greater improvements in both metacognition and empathy compared to those in the comparison group.

Simple mediation analysis further suggested that metacognitive awareness statistically mediated the relationship between the intervention and empathy. Although the direct effect of the intervention on empathy remained significant, the presence of a meaningful indirect effect through metacognition supports the plausibility of a cognitive pathway linking contemplative music practices to socio-emotional outcomes. Importantly, this mediation should be interpreted as a theoretically informed statistical association rather than evidence of temporal or causal sequencing, given the study design and measurement timing.

Taken together, these findings contribute to emerging research on contemplative practices by demonstrating that structured classical music listening, when integrated with mindfulness principles, may foster reflective cognitive monitoring and empathic orientation. By operationalizing Buddhist mindfulness concepts within a replicable music-based program, the study offers a theoretically grounded framework for examining the cognitive and socio-emotional dimensions of contemplative engagement.

Despite methodological limitations—including reliance on self-report measures, a modest sample size, and the absence of longitudinal follow-up—the results provide preliminary evidence supporting the integration of classical music and mindfulness in educational and applied psychology contexts. Future research employing randomized controlled designs, multi-method assessments, and diverse populations will be essential to further clarify the mechanisms and generalizability of these effects.

Overall, the present study provides a conceptually grounded and empirically informed foundation for understanding how classical music meditation may interact with metacognitive and empathic processes, within the limits of the present study design, while highlighting important directions for continued theoretical refinement and methodological advancement.

## Data Availability

The datasets presented in this study can be found in online repositories. The names of the repository/repositories and accession number(s) can be found in the article/Supplementary material.
