# Supplementary material for: Effects of a classical music meditation program on metacognitive awareness and empathy: a simple mediation model informed by Buddhist mindfulness theory
Source: Front Psychol. 2026 Jan 22;16:1713818. doi: 10.3389/fpsyg.2025.1713818 (PMC12874395; doi:10.3389/fpsyg.2025.1713818)
Supplement: Supplementary file 1 [file Table_1.docx]

**Supplementary Table S1. Correlation Matrix of Study Variables**

**Table S1**
Pearson correlation coefficients among classical music meditation participation, metacognitive awareness, and empathy (N = 50).

| Variable | 1 | 2 | 3 |
| --- | --- | --- | --- |
| 1. Classical Music Meditation (Group) | 1 |  |  |
| 2. Metacognitive Awareness | .41** | 1 |  |
| 3. Empathy | .38** | .52** | 1 |

**Note.**
Group was coded as 0 = comparison group, 1 = classical music meditation group.
**p < .01.**

**Supplementary Table S2. A Priori Power Analysis**

**Table S2**
A priori power analysis conducted using G*Power 3.1 for detecting mediation effects.

| Parameter | Value |
| --- | --- |
| Statistical test | Linear multiple regression (fixed model, R² deviation from zero) |
| Effect size (f²) | 0.15 (medium) |
| α error probability | 0.05 |
| Power (1 − β) | 0.80 |
| Number of predictors | 2 |
| Required sample size | 44 |
| Actual sample size | 50 |

**Note.**
The a priori power analysis indicated that a minimum of 44 participants was required to detect a medium mediation effect with adequate statistical power. The final sample size of 50 exceeded this requirement.
